# Clinical outcomes of newly diagnosed primary CNS lymphoma treated with ibrutinib‐based combination therapy: A real‐world experience of off‐label ibrutinib use

**DOI:** 10.1002/cam4.3499

**Published:** 2020-10-17

**Authors:** Feili Chen, Diwen Pang, Hanguo Guo, Qiuxiang Ou, Xue Wu, Xinmiao Jiang, Xiaojuan Wei, Sichu Liu, Ling Huang, Zhanli Liang, Dong Zhou, Wenyu Li

**Affiliations:** ^1^ Lymphoma Division Guangdong Provincial People's Hospital Guangdong Academy of Medical Sciences School of Medicine South China University of Technology Guangzhou China; ^2^ Translational Medicine Research Institute Geneseeq Technology Inc Toronto ON Canada; ^3^ Department of Neurosurgery Guangdong Provincial People's Hospital Guangdong Academy of Medical Sciences Guangzhou China

## Abstract

Ibrutinib‐based combination therapy with high‐dose methotrexate (HD‐MTX) has recently shown clinical activity against relapse/refractory (R/R) primary central nervous system lymphoma (PCNSL). Herein, we report our real‐world experience of treating 11 newly diagnosed PCNSL patients with the ibrutinib/MTX combination. HD‐MTX was given at 3.5 g/m^2^ every 2‐week for eight doses. Ibrutinib was held upon HD‐MTX infusion until clearance and was administered daily post‐induction until disease progression, intolerable toxicity, or death. Nine out of 11 patients completed the induction phase and received ibrutinib as maintenance therapy. An objective response rate (ORR) of 82% (9/11) was observed including complete response (64%) and partial response (18%). The median progression‐free survival (PFS) was 7.4 months while the median overall survival (OS) was not reached. The ibrutinib/MTX combination was well tolerated in these treatment‐naïve PCNSL patients with an acceptable safety profile. Moreover, the longitudinal analysis of cerebrospinal fluid (CSF) circulating tumor DNA (ctDNA) revealed that CSF ctDNA detection was closely associated with tumor response, and sustained tumor responses correlated with the clearance of ctDNA from the CSF. In sum, our data not only demonstrated the clinical benefit of the ibrutinib and HD‐MTX combination regimen in treating newly diagnosed PCNSL patients in a real‐world setting, but also highlighted the significance of liquid biopsy including CSF ctDNA in tracing tumor burden and assessing treatment response.

## Graphical abstract

Our real‐world experience demonstrated the clinical benefit of the ibrutinib‐based combination therapy in treating newly diagnosed PCNSL patients, in complement to the activity of the drug combination reported for relapse/refractory PCNSL. Our data also underscored the clinical significance of liquid biopsy including cerebrospinal fluid ctDNA in tracing tumor burden and evaluating treatment response.

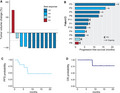

## 1 INTRODUCTION

Primary CNS lymphoma (PCNSL) is a rare subtype of aggressive non‐Hodgkin lymphoma located in the brain, leptomeninges, spinal cord, cerebrospinal fluid, or the vitreoretinal compartment, without evident systemic disease.^1^, ^2^ It accounts for less than 3% of all cases of non‐Hodgkin lymphoma and almost 3% of all primary central nervous system tumors.^1^ High‐dose methotrexate (HD‐MTX) has formed the backbone of the PCNSL treatment.^3^ However, about half of the patients have relapsed/refractory disease.^4^ Moreover, the presence of blood–brain barrier hinders the use of multiple drugs in PCNSL.

Recently, numerous studies have reported the genomic alterations of PCNSL. Myeloid differentiation factor 88 (MYD88) is an adaptor molecule in the Toll‐like receptor and interleukin‐1 receptor signaling pathway. It is implicated in tumorigenesis through activation of the nuclear factor (NF)‐kB signaling pathway.^5^, ^6^ Activated B cell like ‐diffuse large B cell lymphoma (ABC‐DLBCL) is dependent on *MYD88* as reported by Ngo et al.^6^ *MYD88* L265P is the most common *MYD88* mutant variant in systemic DLBCL.^6^ ABC‐DLBCL is the most frequent subtype of PCNSL.^7^ Thus, *MYD88* is frequently mutated in PCNSL.^8^, ^9^, ^10^, ^11^, ^12^ *CD79B* was the second most frequently mutated gene found in PCNSL.^8^, ^9^, ^10^, ^11^, ^12^ *CD79B* is a component of B cell receptor (BCR). Mutations of *CD79B* can promote chronic, active BCR signaling and NF‐κB activation.^13^ It can provide survival signals to the tumor cells.^13^ The presence of *MYD88 L265P* and *CD79B* mutations was also used to classify DLBCLs into different categories.^14^ *CARD11* is a downstream member of BCR pathway. Mutations of *CARD11* may contribute to NF‐κB activation and thereby play a role in the pathogenesis of PCNSL.^15^ *CARD11* mutations have been associated with resistance to single‐agent ibrutinib in several human B cell malignancies.^16^, ^17^

Ibrutinib, the first‐in‐class Bruton tyrosine kinase (BTK) inhibitor, is able to cross the blood–brain barrier.^12^, ^18^ It could mediate signals downstream of *MYD88* and *CD79B*. Therefore, ibrutinib is a promising drug in PCNSL and has shown antitumor in preclinical PCNSL models as well as patients with relapsed/refractory PCNSL.^10^, ^11^, ^12^, ^18^ However, the response to single‐agent ibrutinib has always been incomplete and transient, lasting about 6 months, requiring combination therapy.^10^, ^18^ In a prospective study, researchers combined temozolomide, etoposide, liposomal doxorubicin, dexamethasone, rituximab, and intrathecal cytarabine to treat newly diagnosed and relapsed/refractory PCNSL patients.^11^ The response rate was high. All newly diagnosed PCNSL patients responded to this regimen. However, it was associated with a relatively high treatment‐associated mortality of 17%.^11^ In a phase 1b study, ibrutinib was combined with HD‐MTX and demonstrated an objective response rate of 80% in relapsed/refractory PCNSL patients.^12^ This regimen also has an acceptable safety profile.

Considering the promising results of the phase 1b study in relapsed/refractory CNS lymphoma patients, we adopted the combination therapy of HD‐MTX and ibrutinib in our daily practice. We also explored next‐generation sequencing of circulating tumor DNA (ctDNA) in cerebrospinal fluid (CSF) before and during the treatment. Herein, we retrospectively analyzed the clinicopathologic characteristics, adverse events, and outcomes in newly diagnosed PCNSL patients who were treated with HD‐MTX and ibrutinib combination therapy, and safety profile, treatment response, and genomic biomarkers were reported.

## 2 METHODS

### 2.1 Patient enrolment

From December 2018 to June 2019, a total of 11 newly diagnosed PCNSL patients who were at least 18 years old, subject to radiology test for clinical staging, and received at least two cycles of chemotherapy during treatment, were retrospectively analyzed. None of the patients had a history of other cancers. There was no washout period, but patients received dexamethasone (<10 mg) before starting the trial of the current study medications. Patients did not have HIV/AIDS, except that one patient (P7) had diabetes but no chronical kidney disease. Patients had similar prognosis according to baseline demographic and clinical characteristics. Baseline staging were determined using magnetic resonance imaging (MRI), cerebrospinal fluids analysis, ophthalmologic examination, whole‐body positron emission tomography or enhance contrasted tomography and bone marrow biopsy. This study was approved by the Clinical and Research Ethics Committee of the Guangdong Provincial People's Hospital, Guangzhou, China. All procedures in the present study that involved human participants were performed in accordance with the Declaration of Helsinki (DoH).

### 2.2 Treatment protocol and response assessment

The treatment regimen consisted of a combined induction therapy with HD‐MTX and Ibrutinib, followed by ibrutinib as a maintenance therapy. HD‐MTX was given at 3.5 g/m^2^ every 2 weeks, for a total of eight doses. Ibrutinib dose was 560 mg/d. Ibrutinib was held on days of HD‐MTX infusion and resumed after HD‐MTX clearance. Daily ibrutinib was administered continuously after completion of induction therapy until disease progression, development of intolerable toxicity including severe hepatic, bone marrow, and renal toxicity, or death. Treatment response was evaluated following the international PCNSL Collaborative Group guidelines.^19^ Response to treatment was assessed every two cycle in all CNS compartments using MRI imaging and CSF cytology. Patients’ best response to treatment was recorded for calculating the objective response rate (ORR), which was defined as the proportion of patients with complete response (CR, no contrast enhancing disease) or partial response (PR, 50% or more decrease in enhancement) according to the guideline.^19^ More specifically, total tumor volume was defined as the sum of volumes of maximally six target lesions as measured by the largest longitudinal diameter multiplied by its perpendicular diameter on the same MRI image. A time window period of 3‐day was generally allowed for MRI scans either prior to or after CSF sample collection.

### 2.3 Sample collection and genomic analysis

Cerebrospinal fluids were collected at baseline and on day 15 of cycle 2, 4, 6, 8 (immediately before the initiation of cycle 3, 5, 7, and maintenance phase) and analyzed using capture‐based targeted next‐generation sequencing by gene panels covering lymphoma‐associated genes. Baseline tumor biopsy samples were also subject to sequencing if available. Specifically, mutated *MYD88* was detected in the pretreatment tumor tissue sample of patient P2 using sanger sequencing at the Department of Pathology of the Guangdong Provincial People's Hospital, Guangzhou, China. Genomic analysis followed methods and algorithm as previously described.^20^, ^21^ DLBCL subtype (non‐germinal center B cell (non‐GCB) or germinal center B cell (GCB)) was determined using immunohistochemical staining for CD10, BCL‐6, and MUM‐1, following the Hans classification in the Department of Pathology of the Guangdong Provincial People's Hospital, Guangzhou, China.

### 2.4 Statistics

Statistical software SPSS 21.0 (IBM, Chicago, IL) was used for data analysis. We use descriptive statistics such as means, standard deviations, and medians for continuous variables and proportions to summarize discrete variables. The Kaplan–Meier method was used to calculate all survival end points, which were compared by log‐rank test. A two‐sided *p* < 0.05 indicated a significant difference. Overall survival (OS) was calculated from the date of diagnosis to the time of death from any cause. Progression‐free survival (PFS) was calculated from the start of treatment to the time of disease progression or death due to PCNSL.

## 3 RESULTS

### 3.1 Patients

A total of 11 patients was retrospectively analyzed. Patients’ clinicopathological characteristics are summarized in Table 1. Median age was 56 years (range, 41‐68) and seven patients had an ECOG performance score higher than 2 (Table S1). Four patients were women. Eight patients had lesions in deep areas, including the periventricular tissue, basal ganglia, corpus callosum, brainstem, and/or cerebellum. Eight patients had high cerebrospinal fluid protein concentrations (>450 mg/L) while only one patient had high lactic dehydrogenase serum level (>250 U/L). The International Extranodal Lymphoma Study Group (IELSG) risk score was low (0‐1) for one patient and high (4‐5) for two patients.

**TABLE 1:** Summary of patients’ baseline characteristics

Characteristics	All, N = 11
Age, years	56 (range: 41‐68)
Sex, no. (%)	
Male	7 (63.6)
Female	4 (36.4)
ECOG PS≥2, no. (%)	7 (63.6)
Invasion of deep intracranial areas, no. (%)	8 (72.7)
High CSF protein concentrations (> 450 mg/L), no. (%)	8 (72.7)
High LDH serum level (> 250 U/L), no. (%)	1 (9.1)
IELSG risk score, no. (%)	
Low	1 (9.1)
Intermediate	8 (72.7)
High	2 (18.2)
Time of follow‐up (months)	11.6 ± 5.3

Plus‐minus values are ±SD.

ECOG PS, Eastern Cooperative Oncology Group Performance Score; IELSG, International Extranodal Lymphoma Study Group.

### 3.2 Safety and adverse events

A total of 79 doses of induction therapy and 31 doses of ibrutinib maintenance therapy were given. None of the patients received corticosteroid therapy. The dose of HD‐MTX was not reduced in any of the patients. HD‐MTX was delayed in two patients. One delay was due to grade 3 pneumonia and grade 4 thrombocytopenia and the other was due to grade 3 pneumonia. As shown in Table 2, the most common adverse effect was anemia (100%), followed by hypoalbuminemia (81.8%) and hypokalemia (63.6%). However, these side effects were mild, requiring no further treatment. We observed no treatment‐related death.

**TABLE 2:** Adverse events

Adverse event	Grade 1 or 2	Grade 3	Grade 4	Total (%)
Hematological toxicities				
Leukopenia	2			2 (18.2)
Neutropenia	2			2 (18.2)
Thrombocytopenia			1	1 (9.1)
Anemia	11			11 (100)
Non‐hematological toxicities				
Transaminase increase	2			2 (18.2)
Creatinine increase	1			1 (9.1)
Hypoalbuminemia	9			9 (81.8)
Hypokalemia	7			7 (63.6)
Lung infection		2		2 (18.2)

### 3.3 Treatment duration and response

Nine of 11 patients completed the induction phase of ibrutinib‐based combination therapy (72 delivered out of 72 cycles planned). Two patients did not complete the induction regimen due to disease progression after cycle 4 (P7) and cycle 2 (P8). Nine patients started the maintenance phase of single‐agent ibrutinib. Two patients (P2 and P3) quit the maintenance therapy due to financial difficulty 1 year later of diagnosis. Three patients stopped receiving ibrutinib due to tumor progression (P1, P4, and P11). None of the patients received antifungal prophylaxis treatment.

**FIGURE 1:**
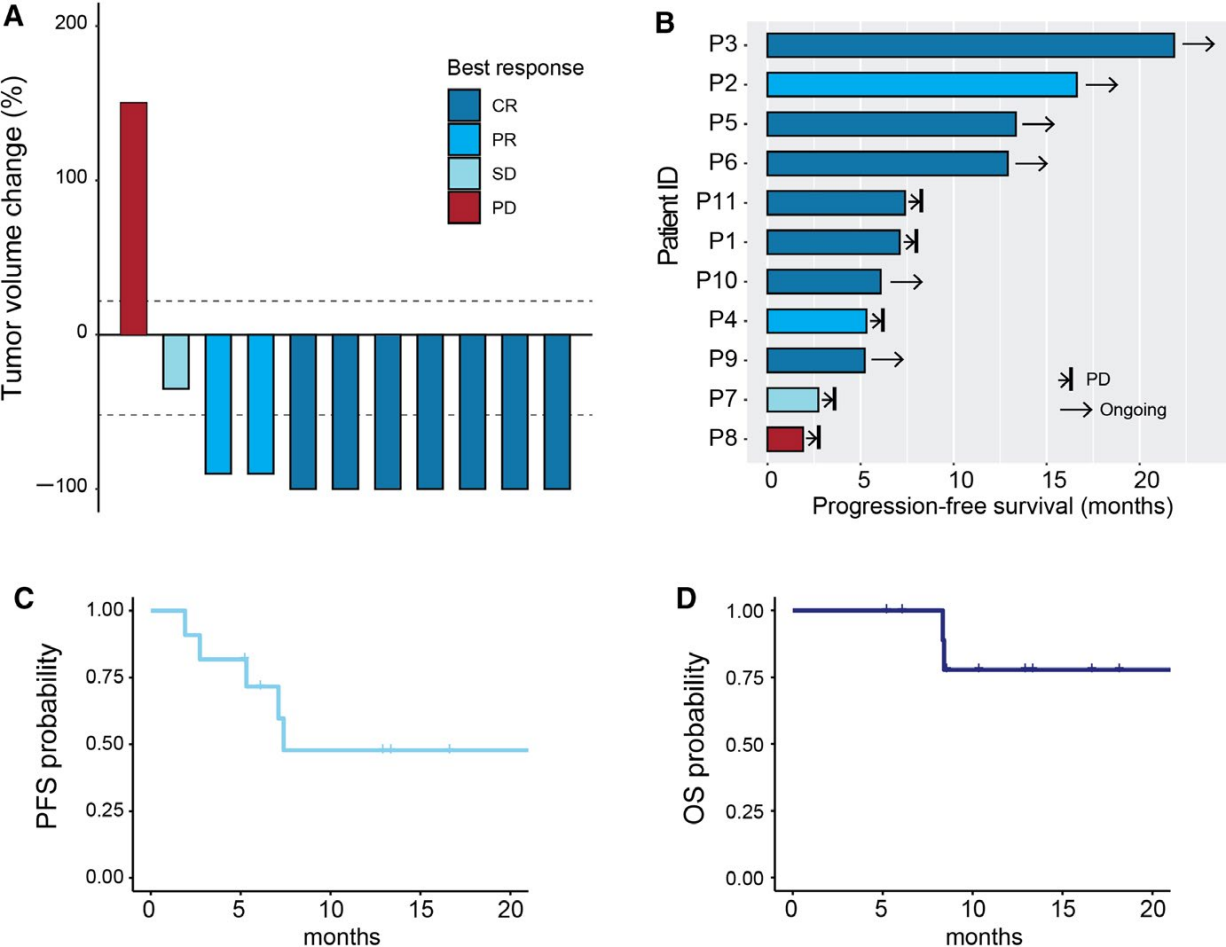
Clinical response to ibrutinib plus HD‐MTX therapy in PCNSL. (A) Best response to therapy. Percentage change of the total tumor volume (see Methods) from baseline was determined by MRI images. (B) Summary of patients’ PFS. (C) Kaplan–Meier for PFS. (D) Kaplan–Meier for OS

The average follow‐up time was 11.6 months (standard deviation = 5.3 months). All patients were evaluated for treatment response, including complete response (CR) for seven patients, partial response (PR) for two patients, disease remained stable in one patient, and progressed disease observed in one patient, demonstrating an objective response rate (ORR) of 81.8% (Figure 1A). The median PFS for the entire cohort was 7.4 months (Figure 1B,C). Two patients (P1 and P8) were deceased upon the time of last follow‐up, while the median OS was not reached for the entire cohort (Figure 1D).

### 3.4 Clinical response and baseline tumor genomic characteristics

**FIGURE 2:**
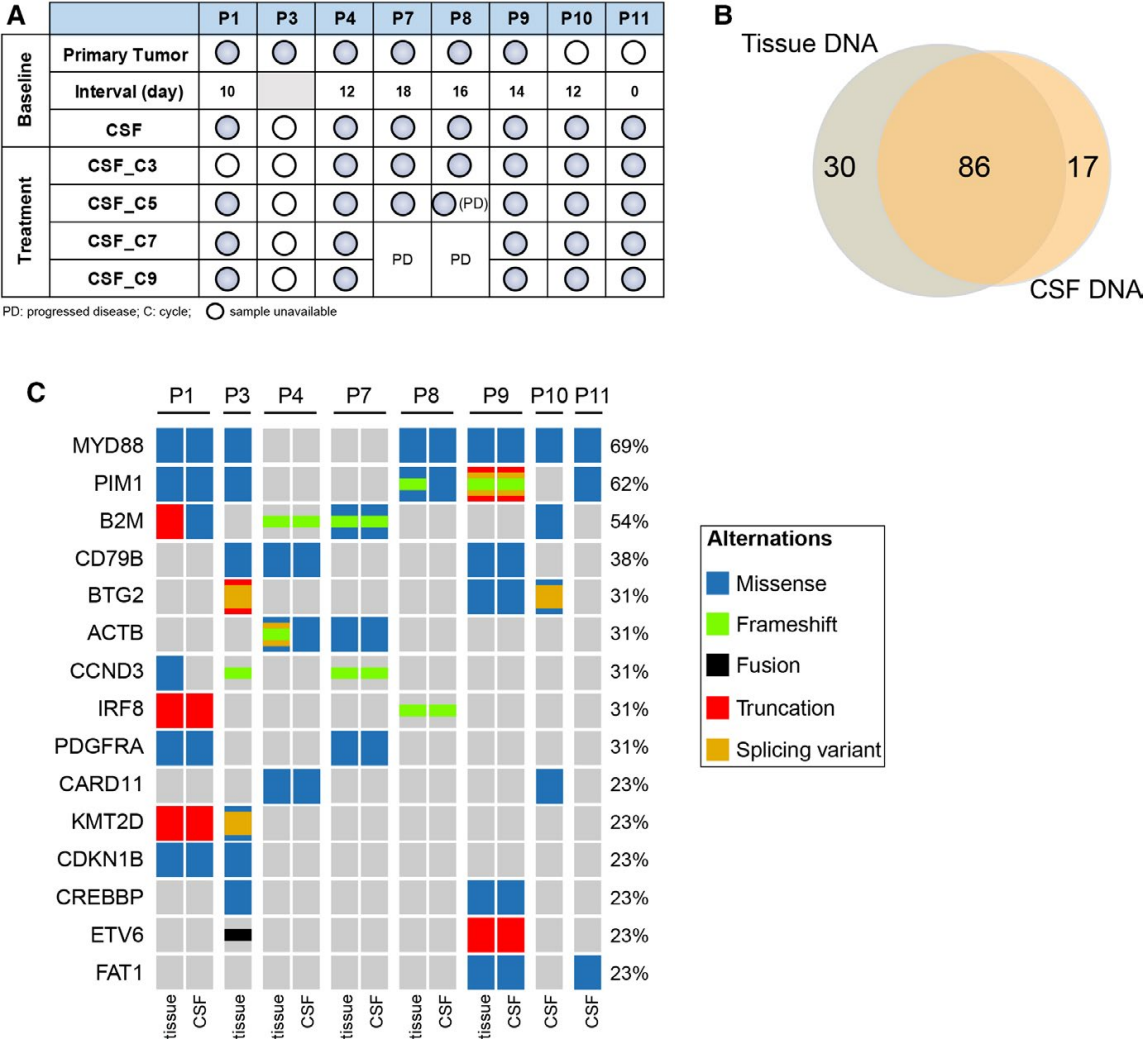
Molecular analysis of tissues or CSF (A) The timeline of sample collection for sequencing. Filled circles indicate CSF samples collected. Empty circles are defined as CSF samples unavailable. The interval indicated the time between the tumor and CSF specimen collection. PD, progressed disease. (B) Concordance between baseline primary tissue and CSF samples for mutation detection. (C) Co‐mutation plot showing genes of which alterations were detected in more than one patient

We next explored the association between treatment response and tumor genomic traits. Baseline tumor biopsy samples were available for six patients, while seven patients had CSF samples available for genomic analysis (Figure 2A). A total of 133 genetic alterations were detected (CSF:103; Tumor:116) and 86 alterations were shared between primary tumor and CSF samples (Figure 2B). *MYD88 L256P* was the most common mutation detected in both CSF and primary tumor samples, followed by mutations of *PIM1* ^22^ and *B2M* ^23^ genes (Figure 2C). Five patients had paired baseline primary tumor and CSF samples, and there was a remarkable concordance (74%, 86/116) between baseline CSF and primary tumor specimens for mutations identified.

Furthermore, eight of 11 patients had non‐GCB disease and seven (87.5%) responded to the ibrutinib‐based regimen. Two patients had GCB disease and one patient who had *MYD88* L256P mutation achieved CR on the ibrutinib‐based regimen. Eight of nine patients had mutations in at least one gene involved in the BCR pathway, including *MYD88* (7/9, 77.8%), *CD79B* (3/9, 33.3%), and *CARD11* (3/9, 33.3%) (Table 3). Three patients (P3, P4, and P10) who contained *CARD11* mutations also responded to ibrutinib‐based treatment (Figure 1B).

**TABLE 3:** Mutations in baseline primary tumor tissue or CSF specimens

ID	COO subtype	Best response (months)	*MYD88*	*CD79B*	*CARD11*
P1	Non‐GCB	CR (7.1)	L265P (T, C)	WT (T, C)	WT (T, C)
P2	Non‐GCB	PR (16.6)^a^	L265P (T)	NA	NA
P3	GCB	CR (21.8)^a^	L265P (T)	A30V, Y197S (T)	WT (T)
P4	Non‐GCB	PR (5.3)	WT (T, C)	A30V (T, C)	F115I (T, C)
P5	Non‐GCB	CR (13.3)^a^	NA	NA	NA
P6	Non‐GCB	CR (12.9)^a^	NA	NA	NA
P7	GCB	SD (2.7)	WT (T, C)	NA	NA
P8	Non‐GCB	PD (1.9)	L265P (T, C)	WT (T, C)	WT (T, C)
P9	Non‐GCB	CR (5.2)^a^	L265P (T, C)	Y197H (T, C)	WT (T, C)
P10	Non‐GCB	CR (6.1)^a^	L265P (C)	WT (C)	M360T (C)
P11	NA	CR (7.4)	L265P (C)	NA	NA

Notes

(C), CSF; (T), archival formalin‐fixed paraffine‐embedded tissue.

COO, Cell of Origin; GCB, Germinal center B cell; NA, not available; WT, wild‐type.

a Still in remission.

### 3.5 Disease surveillance through cerebrospinal fluid ctDNA

**FIGURE 3:**
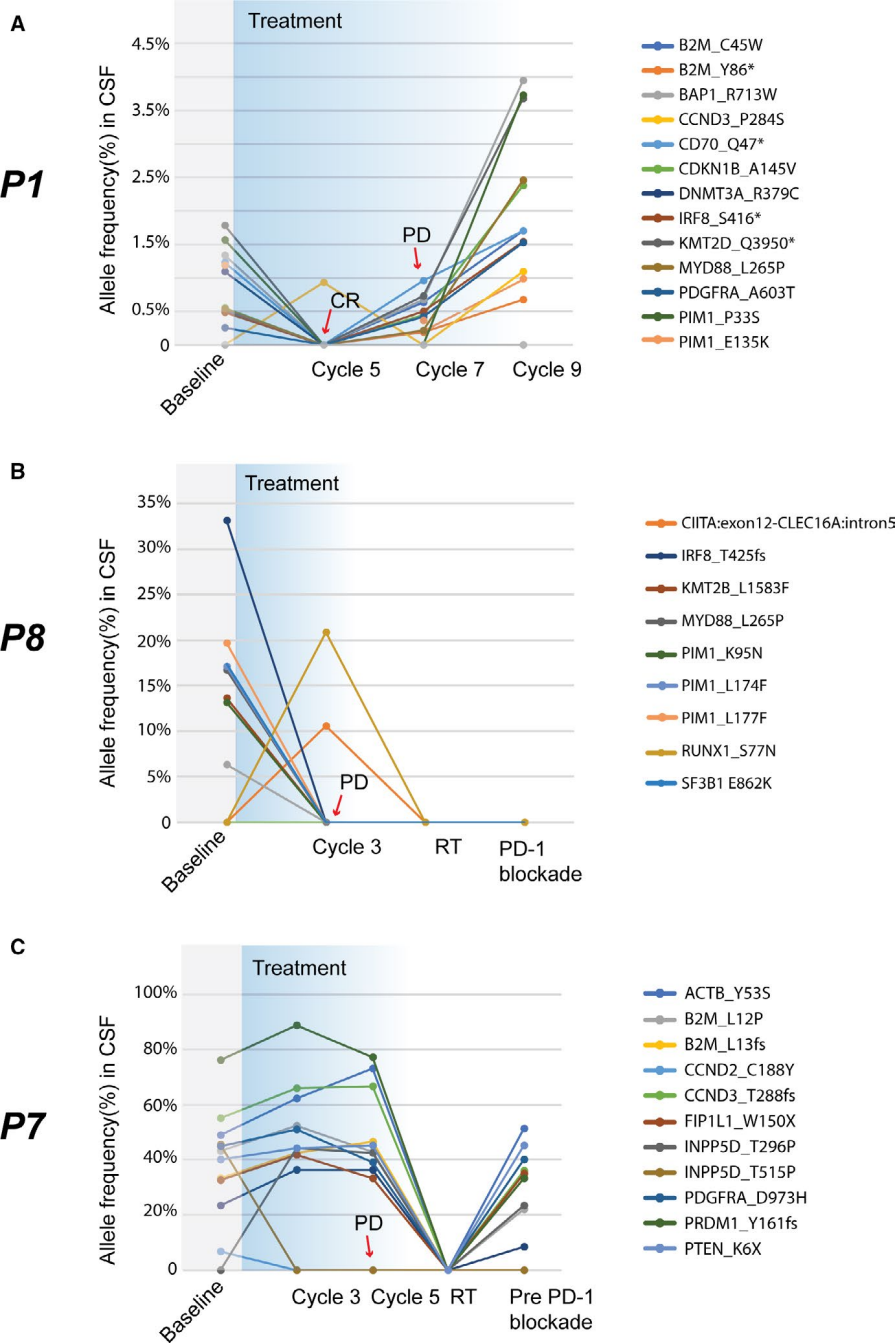
Disease monitoring through CSF ctDNA on therapy. (A) Patient P1 had persistent tumor‐specific alterations in the CSF and suffered from disease progression 3 months after completion of induction phase; (B) Patient P8 got *MYD88* L256P and *CIITA* mutation at diagnosis and received ibrutinib‐based regimen. She experienced disease progression after cycle 2 with the disappearance of *MYD88* L256P mutation, but the *CIITA* mutation was identified. (C) Patient P7 got persistent tumor‐specific alterations in CSF and progressed soon. CR, complete response; PD, progressed disease; RT, radiotherapy. Alterations were indicated by different colors

For seven patients, CSF samples were collected at baseline, immediately before cycle 3 (C3), cycle 5 (C5), cycle 7 (C7), and maintenance phase (C9). Three patients had a complete radiographic response to the treatment and CSF mutant allele frequency dramatically decreased. Patient P4 demonstrated a partial radiographic response (−90% by MRI) with the clearance of ctDNA in the CSF specimen. However, he progressed 1 month after ibrutinib maintenance. One patient (P1) achieved complete radiographic response to the treatment and negative CSF cytology results, consistent with a marked decrease of CSF mutation AFs (Figure 3A). However, disease progressed 3 months after the induction phase, in line with the recurrence of somatic mutations in the CSF sample (Figure 3A). Patient P8 was identified of *MYD88* L256P and *CIITA* fusion at diagnosis and received ibrutinib‐based regimen (Figure 3B). CIITA is a transactivator of major histocompatibility complex (MHC) class II.^24^ The allele frequency of *MYD88* L256P dramatically reduced prior to disease progression (PD) at the onset of C3, while the *CIITA* fusion sustained (Figure 3B). The patient then received whole‐brain radiotherapy (WBRT, 30Gy) and PD‐1 inhibitor treatment followed by the clearance of *CIITA* mutation. She was alive until the time of the last follow‐up. In addition, patient P7 remained stable disease (SD) until C5 and mutations were continuously detected in CSF samples during the treatment (Figure 3C). He then received whole‐brain radiotherapy (WBRT) upon disease progression followed by undetectable CSF ctDNA. However, he relapsed soon with the recurrence of CSF ctDNA (Figure 3C). He then was treated with PD‐1 inhibitors.

## 4 DISCUSSION

This case series found that the sequential use of ibrutinib with HD‐MTX was well tolerated as first‐line therapy in PCNSL. Nine patients went on to maintenance ibrutinib while the other two patients stopped the therapy due to progressed disease. All eleven patients were included for the evaluation of PFS and OS. We observed no dose reduction and treatment‐related death. Although all suffered from anemia, no patient required infusion of blood cells. Two patients got grade 3 pneumonia causing delay of HD‐MTX. One patient got grade 4 thrombocytopenia without bleeding.

Treatment options for PCNSL has evolved during the last few decades, however, no uniform consensus on the standard therapy currently exists. HD‐MTX is the backbone of current chemotherapy regimens for PCNSL patients.^3^ Polychemotherapy regimens including rituximab demonstrated an ORR of 35%–74%.^25^ More specifically, according to Kasenda et al.,^26^ approximately 68% (CR +PR) of PCNSL patients who were older than 60 years responded to HD‐MTX‐based first‐line chemotherapy, with median PFSs varying between different treatment regimens (range: 4‐24 months). The HD‐MTX‐based treatment was also independently associated with improved OS compared with therapies without HD‐MTX or whole‐brain radiotherapy (WBRT) monotherapy.^26^ In this study, the ibrutinib/HD‐MTX combination regimen showed good antitumor activity with an ORR being 81.8%, higher than using HD‐MTX‐based chemotherapy alone. We also observed CRs in patients harboring *CARD11* mutation which would be predicted to respond poorly to single‐agent ibrutinib.^16^, ^17^ Patient P3 has stopped ibrutinib for more than 1 year and is still in complete remission, indicating the probability of long‐time remission. However, we were unable to explore to what extent ibrutinib increased the antitumor activity of HD‐MTX in newly diagnosed PCNSL or vice versa due to the non‐randomized design of our study, the limited *N* size, and the relatively short follow‐up. Larger‐scale prospective cohort studies and longer follow‐up may be warranted to validate these results in the future.

Three of the nine responding patients relapsed soon after the completion of HD‐MTX, indicating the limitation of ibrutinib maintenance alone. Autologous stem transplantation has been demonstrated to be an effective consolidation strategy in PCNSL.^27^ Thus, patients fit for stem transplantation should proceed to autologous stem transplantation. Lenalidomide is an immunomodulatory agent that downregulates *IRF4* downstream of BCR and MYD88 signaling.^28^ In preclinical studies, lenalidomide increased the antitumor activity of ibrutinib in diffuse large B cell lymphoma model.^29^ Moreover, in a prospective clinical trial, combination of lenalidomide, ibrutinib, and rituximab has promising antitumor activity in r/r diffuse large B cell lymphoma, especially in patients with non‐GCB subtype.^30^ Lenalidomide also has antitumor activity in PCNSL.^31^, ^32^ The pathological subtype of most PCNSL patients is non‐GCB subtype, indicating the promising future of combing lenalidomide and ibrutinib as maintenance in PCNSL. For patients unfit for autologous stem transplantation, use of lenalidomide plus ibrutinib as maintenance might be promising. Additionally, Nayak et al. reported that PD‐1 blockade with nivolumab demonstrated activity in a small cohort of r/r PCNSL and primary testicular lymphoma patients who had *PD*‐*1* copy number gain, supporting further investigation of PD‐1 blockade in these diseases.^33^

Another controversial issue is that a positive effect of the use of rituximab on the outcome in PCNSL patients remains uncertain due to its poor ability to cross blood–brain barrier. In another prospective study with relatively larger sample size (*N* = 200), patients failed to benefit from the combination of rituximab with methotrexate, carmustine, teniposide, and prednisone.^34^ However, a prior retrospective study showed that the addition of rituximab to HD‐MTX and temozolomide conferred significant clinical benefits.^35^ Moreover, Grommes et al found that addition of rituximab to HD‐MTX and ibrutinib could result a higher CR rate in relapsed/refractory CNS lymphoma (56% vs. 33%).^12^ Thus, combination of rituximab, ibrutinib, and HD‐MTX might have a promising antitumor activity in PCNSL. Not denying the fact that economic burden was one of the major factors to account for during treatment decision‐making, patients did not receive rituximab in this study. Ibrutinib plus HD‐MTX with rituximab in newly diagnosed PCNSL patients is currently under investigation in our institution.

Our findings also demonstrated that CSF liquid biopsies profiling using next‐generation sequencing was feasible in PCNSL. None of our patients had a positive CSF cytology at baseline. However, for seven patients who had CSF available for genetic analysis, more than one mutation was detected in CSF. Thus, CSF ctDNA detection tends to be more sensitive than the conventional CSF cytology analysis. In this study, we found that CSF mutations profiles were high concordant with tumor mutation profiles, and therefore, sequential detection of ctDNA in CSF might help us to modify the treatment protocol and evaluate the response.

## 5 CONCLUSIONS

In conclusion, our real‐world experience demonstrated that the ibrutinib and HD‐MTX combination regimen brought great clinical benefits in newly diagnosed PCNSL patients, in complement to its activity in r/r PCNSL patients. Our data also highlighted the clinical significance of liquid biopsy including CSF ctDNA in disease surveillance and treatment response monitoring. However, further research with larger patient cohort size and longer follow‐up time is required to validate our findings. In addition, it is of urgent need to investigate the resistance mechanisms to the ibrutinib‐based therapy in PCNSL patients upon tumor progression.

## CONFLICT OF INTEREST

Drs. Qiuxiang Ou and Xue Wu are employees of Geneseeq Technology Inc., Toronto, Canada. The remaining authors have nothing to declare.

## AUTHORS’ CONTRIBUTIONS

FC, DP, and HG conceived and designed the study. FC, DP, HG, XJ, and XJW collected the data. FC, QO and XW analyzed the data. SL, LH, and ZL provided the resources for the study. DZ and WL supervised the study. FC, DP, HG, and QO wrote the manuscript. All authors read and approved the final manuscript prior to submission.

## ETHICS APPROVAL AND CONSENT TO PARTICIPATE

This study was approved by Guangdong Provincial People's Hospital and the Institutional Review Board/Ethics Committee of Guangdong Provincial People's Hospital. All patients provided informed written consent to participate prior to sample collection.

## CONSENT TO PUBLICATION

Written consent was collected from each patient for future data publication.

## Supporting information

Table S1

## Data Availability

The authors confirm that the data supporting the findings of this study are available within the article.

## References

[1] Chukwueke UN , Nayak L . Central nervous system lymphoma. Hematol Oncol Clin North Am.2020;33(4):597‐611. 10.1016/j.hoc.2019.03.008 31229157

[2] Ferreri AJM . Therapy of primary CNS lymphoma: role of intensity, radiation, and novel agents. Hematol Am Soc Hematol Education Program. 2017;2017(1):565‐577. 10.1182/asheducation-2017.1.565 PMC6142584 29222306

[3] Ferreri AJM , Reni M , Foppoli M , et al. High‐dose cytarabine plus high‐dose methotrexate versus high‐dose methotrexate alone in patients with primary CNS lymphoma: a randomised phase 2 trial. Lancet. 2009;374(9700):1512‐1520. 19767089 10.1016/S0140-6736(09)61416-1

[4] Langner‐Lemercier S , Houillier C , Soussain C , et al. Primary CNS lymphoma at first relapse/progression: characteristics, management, and outcome of 256 patients from the French LOC network. Neuro Oncol. 2016;18(9):1297‐1303. 26951382 10.1093/neuonc/now033 PMC4998995

[5] Jeelall YS , Horikawa K . Oncogenic MYD88 mutation drives Toll pathway to lymphoma. Immunol Cell Biol. 2011;89(6):659‐660. 21519346 10.1038/icb.2011.31

[6] Ngo VN , Young RM , Schmitz R , et al. Oncogenically active MYD88 mutations in human lymphoma. Nature. 2011;470(7332):​115‐119. 10.1038/nature09671 PMC5024568 21179087

[7] Hilal T , Maguire A , Kosiorek HE , Rimsza LM , Rosenthal AC . Clinical features and cell of origin subtyping using gene expression profiling in HIV‐negative patients with primary central nervous system lymphoma. Leuk Lymphoma. 2019;60(14):3581‐3583. 31246148 10.1080/10428194.2019.1633637

[8] Braggio E , Van Wier S , Ojha J , et al. Genome‐wide analysis uncovers novel recurrent alterations in primary central nervous system lymphomas. Clin Cancer Res. 2015;21(17):3986‐3994. 25991819 10.1158/1078-0432.CCR-14-2116 PMC4558226

[9] Nakamura T , Tateishi K , Niwa T , et al. Recurrent mutations of CD79B and MYD88 are the hallmark of primary central nervous system lymphomas. Neuropathol Appl Neurobiol. 2016;42(3):279‐290. 26111727 10.1111/nan.12259

[10] Grommes C , Pastore A , Palaskas N , et al. Ibrutinib unmasks critical role of bruton tyrosine kinase in primary CNS lymphoma. Cancer Discov. 2017;7(9):1018‐1029. 28619981 10.1158/2159-8290.CD-17-0613 PMC5581705

[11] Lionakis MS , Dunleavy K , Roschewski M , et al. Inhibition of B cell receptor signaling by ibrutinib in primary CNS lymphoma. Cancer Cell. 2017;31(6):833‐43 e5. 28552327 10.1016/j.ccell.2017.04.012 PMC5571650

[12] Grommes C , Tang SS , Wolfe J , et al. Phase 1b trial of an ibrutinib‐based combination therapy in recurrent/refractory CNS lymphoma. Blood. 2019;133(5):436‐445. 30567753 10.1182/blood-2018-09-875732 PMC6356986

[13] Young RM , Shaffer AL 3rd , Phelan JD , Staudt LM . B‐cell receptor signaling in diffuse large B‐cell lymphoma. Semin Hematol. 2015;52(2):77‐85. 25805587 10.1053/j.seminhematol.2015.01.008 PMC4374122

[14] Schmitz R , Wright GW , Huang DW , et al. Genetics and pathogenesis of diffuse large B‐cell lymphoma. N Engl J Med. 2018;378(15):1396‐1407. 29641966 10.1056/NEJMoa1801445 PMC6010183

[15] Montesinos‐Rongen M , Schmitz R , Brunn A , et al. Mutations of CARD11 but not TNFAIP3 may activate the NF‐kappaB pathway in primary CNS lymphoma. Acta Neuropathol. 2010;120(4):529‐535. 20544211 10.1007/s00401-010-0709-7

[16] Woyach JA , Furman RR , Liu T‐M , et al. Resistance mechanisms for the Bruton's tyrosine kinase inhibitor ibrutinib. N Engl J Med. 2014;370(24):2286‐2294. 24869598 10.1056/NEJMoa1400029 PMC4144824

[17] Wilson WH , Young RM , Schmitz R , et al. Targeting B cell receptor signaling with ibrutinib in diffuse large B cell lymphoma. Nat Med. 2015;21(8):922‐926. 26193343 10.1038/nm.3884 PMC8372245

[18] Soussain C , Choquet S , Blonski M , et al. Ibrutinib monotherapy for relapse or refractory primary CNS lymphoma and primary vitreoretinal lymphoma: Final analysis of the phase II ‘proof‐of-concept’ iLOC study by the Lymphoma study association (LYSA) and the French oculo‐cerebral lymphoma (LOC) network. Eur J Cancer. 2019;117:121‐130. 31279304 10.1016/j.ejca.2019.05.024

[19] Batchelor TT . Primary central nervous system lymphoma. Hematology Am Soc Hematol Educ Program. 2016;2016(1):379‐385. 27913504 10.1182/asheducation-2016.1.379 PMC6142465

[20] Shu Y , Wu X , Tong X , et al. Circulating tumor DNA mutation profiling by targeted next generation sequencing provides guidance for personalized treatments in multiple cancer types. Sci Rep. 2017;7(1):583. 28373672 10.1038/s41598-017-00520-1 PMC5428730

[21] Yang Z , Yang N , Ou Q , et al. Investigating novel resistance mechanisms to third‐generation EGFR tyrosine kinase inhibitor osimertinib in non‐small cell lung cancer patients. Clin Cancer Res. 2018;24(13):3097‐3107. 29506987 10.1158/1078-0432.CCR-17-2310

[22] Nijland M , Seitz A , Terpstra M , et al. Mutational evolution in relapsed diffuse large B‐Cell lymphoma. Cancers (Basel). 2018;10(11):459. 10.3390/cancers10110459 PMC6265691 30463380

[23] Challa‐Malladi M , Lieu YK , Califano O , et al. Combined genetic inactivation of beta2‐Microglobulin and CD58 reveals frequent escape from immune recognition in diffuse large B cell lymphoma. Cancer Cell. 2011;20(6):728‐740. 22137796 10.1016/j.ccr.2011.11.006 PMC3660995

[24] Steidl C , Shah SP , Woolcock BW , et al. MHC class II transactivator CIITA is a recurrent gene fusion partner in lymphoid cancers. Nature. 2011;471(7338):377‐381. 21368758 10.1038/nature09754 PMC3902849

[25] Mendez JS , Grommes C . Treatment of primary central nervous system lymphoma: from chemotherapy to small molecules. Am Soc Clin Oncol Educ Book. 2018;38:604‐615. 30231317 10.1200/EDBK_200829

[26] Kasenda B , Ferreri A , Marturano E , et al. First‐line treatment and outcome of elderly patients with primary central nervous system lymphoma (PCNSL)–a systematic review and individual patient data meta‐analysis. Ann Oncol. 2015;26(7):1305‐1313. 25701456 10.1093/annonc/mdv076 PMC4735103

[27] Ferreri AJ , Illerhaus G . The role of autologous stem cell transplantation in primary central nervous system lymphoma. Blood. 2016;127(13):1642‐1649. 26834241 10.1182/blood-2015-10-636340

[28] Morabito F , Skafi M , Recchia AG , et al. Lenalidomide for the treatment of mantle cell lymphoma. Expert Opin Pharmacother. 2019;20(5):487‐494. 30608891 10.1080/14656566.2018.1561865

[29] Yang Y , Shaffer A , Emre N , et al. Exploiting synthetic lethality for the therapy of ABC diffuse large B cell lymphoma. Cancer Cell. 2012;21(6):723‐737. 22698399 10.1016/j.ccr.2012.05.024 PMC4059833

[30] Goy A , Ramchandren R , Ghosh N , et al. Ibrutinib plus lenalidomide and rituximab has promising activity in relapsed/refractory non‐germinal center B‐cell-like DLBCL. Blood. 2019;134(13):1024‐1036. 31331917 10.1182/blood.2018891598 PMC6764267

[31] Rubenstein JL , Geng H , Fraser EJ , et al. Phase 1 investigation of lenalidomide/rituximab plus outcomes of lenalidomide maintenance in relapsed CNS lymphoma. Blood Adv. 2018;2(13):1595‐1607. 29986852 10.1182/bloodadvances.2017014845 PMC6039666

[32] Ghesquieres H , Chevrier M , Laadhari M , et al. Lenalidomide in combination with intravenous rituximab (REVRI) in relapsed/refractory primary CNS lymphoma or primary intraocular lymphoma: a multicenter prospective ‘proof of concept’ phase II study of the French Oculo‐Cerebral lymphoma (LOC) Network and the Lymphoma Study Association (LYSA)dagger. Ann Oncol. 2019;30(4):621‐628. 30698644 10.1093/annonc/mdz032

[33] Nayak L , Iwamoto FM , LaCasce A , et al. PD‐1 blockade with nivolumab in relapsed/refractory primary central nervous system and testicular lymphoma. Blood. 2017;129(23):3071‐3073. 28356247 10.1182/blood-2017-01-764209 PMC5766844

[34] Bromberg JEC , Issa S , Bakunina K , et al. Rituximab in patients with primary CNS lymphoma (HOVON 105/ALLG NHL 24): a randomised, open‐label, phase 3 intergroup study. Lancet Oncol. 2019;20(2):216‐228. 30630772 10.1016/S1470-2045(18)30747-2

[35] Mao C , Chen F , Li Y , et al. Characteristics and Outcomes of Primary Central Nervous System Lymphoma: A Retrospective Study of 91 Cases in a Chinese Population. World Neurosurg. 2019;123:e15‐e24. 30326304 10.1016/j.wneu.2018.10.034

