# Supplementary material for: Clinical outcomes of newly diagnosed primary CNS lymphoma treated with ibrutinib‐based combination therapy: A real‐world experience of off‐label ibrutinib use
Source: Cancer Med. 2020 Oct 17;9(22):8676–84. doi: 10.1002/cam4.3499 (PMC7666749; doi:10.1002/cam4.3499)
Supplement: Supplementary file 1 — Table S1 [file CAM4-9-8676-s001.docx]

**Supplementary Table S1**. Patients' demographic and clinical data

| **ID** | **Gender** | **Age** | **ECOG** | **IELSG score** | **Histology** | **Follow-up time** | **PFS** | **OS** | **Best response** | **Tumor volume change** |
| --- | --- | --- | --- | --- | --- | --- | --- | --- | --- | --- |
| P1 | M | 56 | 3 | 2 | non-GCB | 8.41 | 7.1 | 8.41 | CR | -100% |
| P2 | M | 54 | 2 | 2 | non-GCB | 16.62 | 16.62 | 16.62 | PR | -90% |
| P3 | M | 60 | 3 | 2 | GCB | 21.85 | 21.85 | 21.85 | CR | -100% |
| P4 | M | 53 | 2 | 2 | non-GCB | 8.54 | 5.32 | 8.54 | PR | -90% |
| P5 | F | 44 | 2 | 3 | non-GCB | 13.34 | 13.34 | 13.34 | CR | -100% |
| P6 | F | 59 | 3 | 1 | non-GCB | 12.91 | 12.91 | 12.91 | CR | -100% |
| P7 | M | 68 | 3 | 3 | GCB | 8.34 | 2.73 | 8.34 | SD | -35% |
| P8 | F | 50 | 1 | 4 | non-GCB | 10.35 | 1.91 | 10.35 | PD | 150% |
| P9 | F | 68 | 1 | 3 | non-GCB | 5.22 | 5.22 | 5.22 | CR | -100% |
| P10 | M | 41 | 1 | 3 | non-GCB | 6.08 | 6.08 | 6.08 | CR | -100% |
| P11 | M | 57 | 1 | 4 | NA | 18.14 | 7.39 | 18.14 | CR | -100% |

ECOG: Eastern Cooperative Oncology Group Performance Status; IELSG: International extranodal lymphoma study group score
